# Supplementary material for: In situ micropillar compression of an anisotropic metal-organic framework single crystal
Source: Commun Chem. 2023 Apr 4;6:63. doi: 10.1038/s42004-023-00858-w (PMC10073295; doi:10.1038/s42004-023-00858-w)
Supplement: Supplementary file 2 — Supplemental Information [file 42004_2023_858_MOESM2_ESM.pdf]

Supplementary Information

for

In situ micropillar compression of an anisotropic metal-organic framework single crystal

Zhixin Zeng,[1] Yuan Xiao,[2] Jeffrey M. Wheeler,[2] and Jin-Chong Tan[*,1]

[1]Multifunctional Materials & Composites (MMC) Laboratory, Department of Engineering Science, University of Oxford, Parks Road, Oxford, OX1 3PJ, U.K.

[2]Laboratory for Nanometallurgy, Department of Materials, ETH Zurich, Vladimir-Prelog-Weg 5, HCI G 503, 8093 Zürich, Switzerland.

[*]*Corresponding author's e-mail: jin-chong.tan@eng.ox.ac.uk*

Table of Contents

1. Supplementary Methods 3

2. Supplementary Note 1: Buckling Strength / Pillar Stability / Euler's Critical Load 6

3. Uniaxial Micropillar Compression Videos and Micropillar Splitting Videos 7

4. Instrumented Nanoindentation Results 8

5. Supplementary Note 2: Yield Stress (*Y*) Determination 9

6. Gamma Coefficient and *H/E* vs. *Y/E Ratios* 11

Supplementary References...12

1. Supplementary Methods

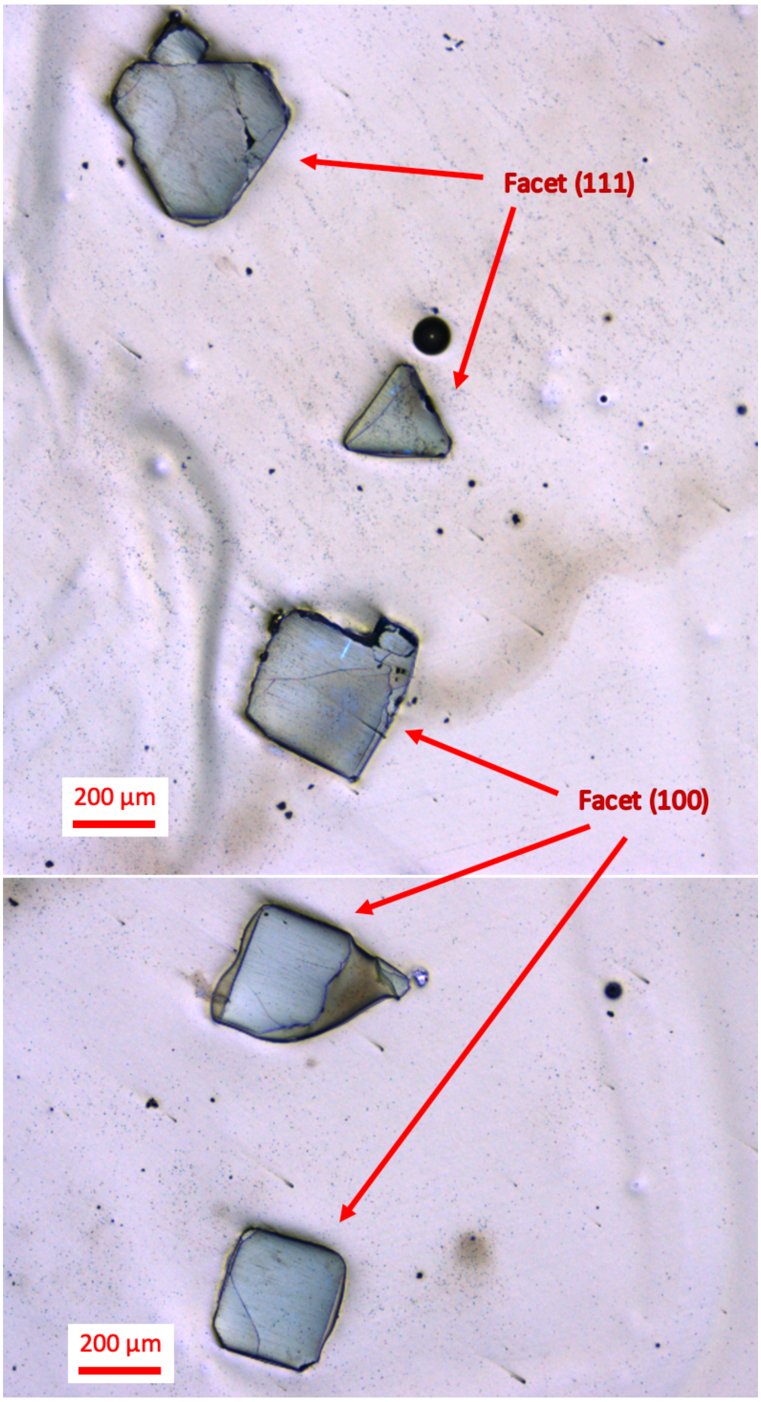

Supplementary Figure 1. Single crystals of HKUST-1 oriented normal to the (111) and (100) facets, mounted on epoxy, carefully ground and polished to expose the smooth surfaces for FIB milling (see Supplementary Supplementary Figure 2).

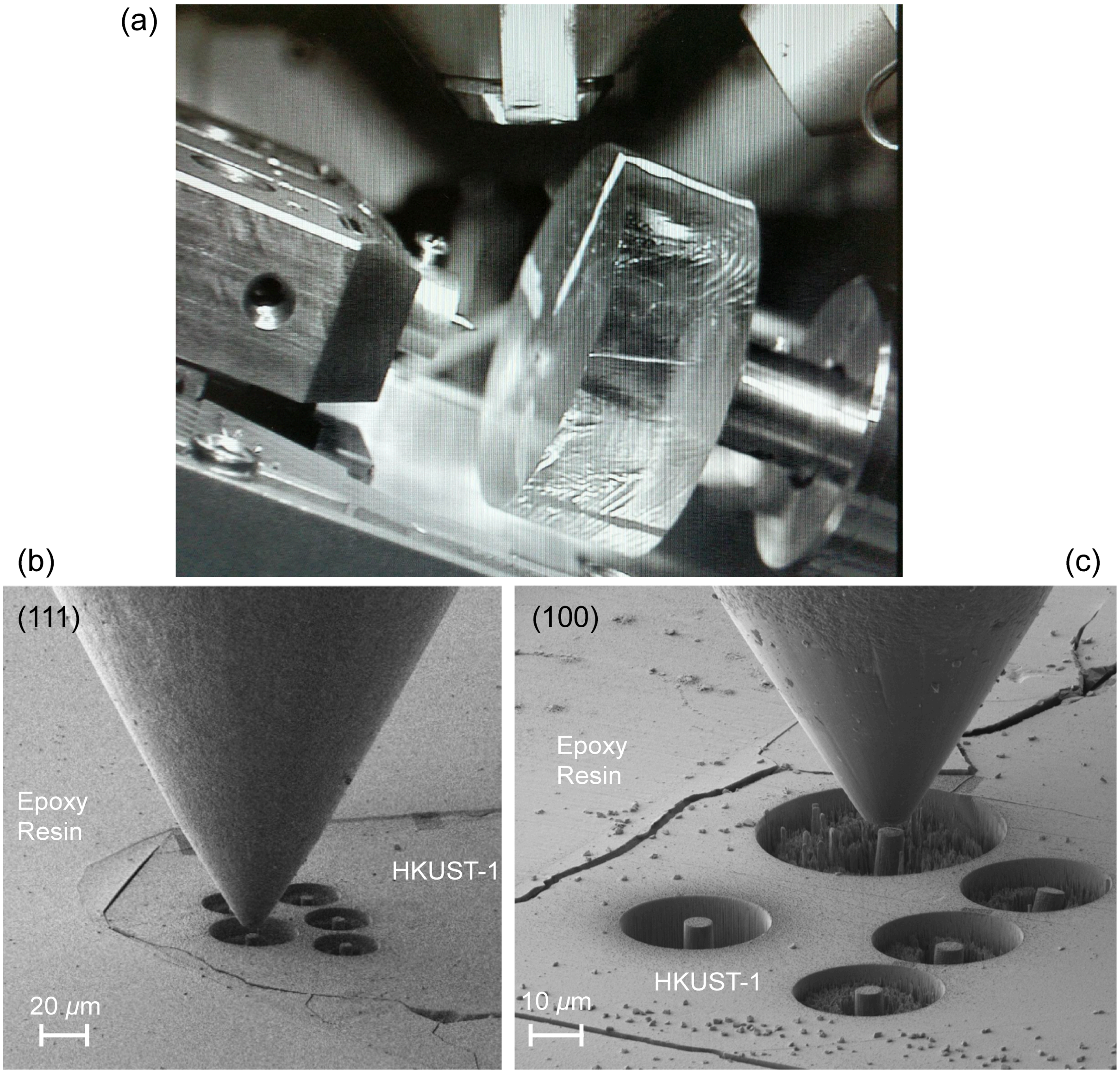

Supplementary Figure 2. Optical and SEM mages of the specimens. (a) Optical image of the HKUST-1 specimen mounted on the Alemnis indenter positioned in a scanning electron microscope (SEM), in which the crystals were embedded on the surface of epoxy resin stub (thickness ~ 1 cm). *In situ* SEM images of the micropillars of HKUST-1 milled by focused ion beam on the (b) (111)- and (c) (100)-crystal facets, respectively. Note that the two pillars located underneath the flat-punch indenter had just been tested.

Supplementary Table 1. Diameters and heights of the micropillar specimens of HKUST-1.

**Crystal Orientation**	**Type of Test**	**Diameter, *D* (μm)**	**Height, *L* (μm)**	***L*/*D* ratio**
(100)	Compression	4.62	14.44	3.13
	Compression	4.92	15.47	3.14
	Compression	4.52	16.27	3.60
	Splitting	5.39	14.80	2.75
	Splitting	5.05	16.17	3.20
(111)	Compression	4.93	15.40	3.12
	Compression	4.76	14.00	2.94
	Compression	5.28	14.40	2.73
	Splitting	5.26	13.76	2.62
	Splitting	5.22	16.17	3.10

2. Supplementary Note 1: Buckling Strength / Pillar Stability / Euler's Critical Load

(a) Boundary Condition (BC) Type 1 – Bottom of the pillar is fixed but the top surface is free, the following supplementary equation gives the Euler's critical load (P_E):

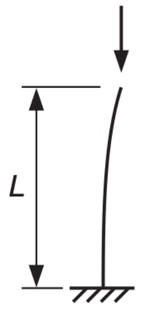

$$P_E = 0.25\frac{\pi^2 EI}{L^2}$$

… Supplementary Equation (1)

(b) Boundary Condition Type 2 – Bottom of the pillar is fixed and there is a mutual movement between the load cell and the top surface of the pillar, the Euler's critical load can be obtained using the following equation:

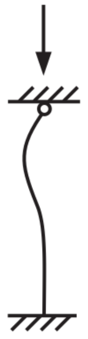

$$P_E = 2.05\frac{\pi^2 EI}{L^2}$$

… Supplementary Equation (2)

where E is the Young's modulus and I is the second moment of area (for a circular section, $I = \frac{\pi r^4}{4}$); L is the height of the pillar.[2]

3. Uniaxial Micropillar Compression Videos and Micropillar Splitting Videos

Supplementary Table 2. Video recordings obtained during micropillar compression (abbreviated as ‘comp’) and splitting tests (abbreviated as ‘split’).

**Crystal Orientation**	**Supplementary Movie #**	**Micropillar *in situ* tests**	**Compression** (*Video frames accelerated* ~15×)	**Splitting** *(Video frames accelerated* ~20×)
(100)	1	(100) P1 comp.	Pillar #1	-
	2	(100) P2 comp.	Pillar #2	-
	3	(100) P3 comp.	Pillar #3	-
	4	(100) P4 split	-	Pillar #4
	5	(100) P5 split	-	Pillar #5
(111)	6	(111) P6 comp.	Pillar #6	-
	7	(111) P7 comp.	Pillar #7	-
	8	(111) P8 comp.	Pillar #8	-
	9	(111) P9 split	-	Pillar #9
	10	(111) P10 split	-	Pillar #10

4. Instrumented Nanoindentation Results

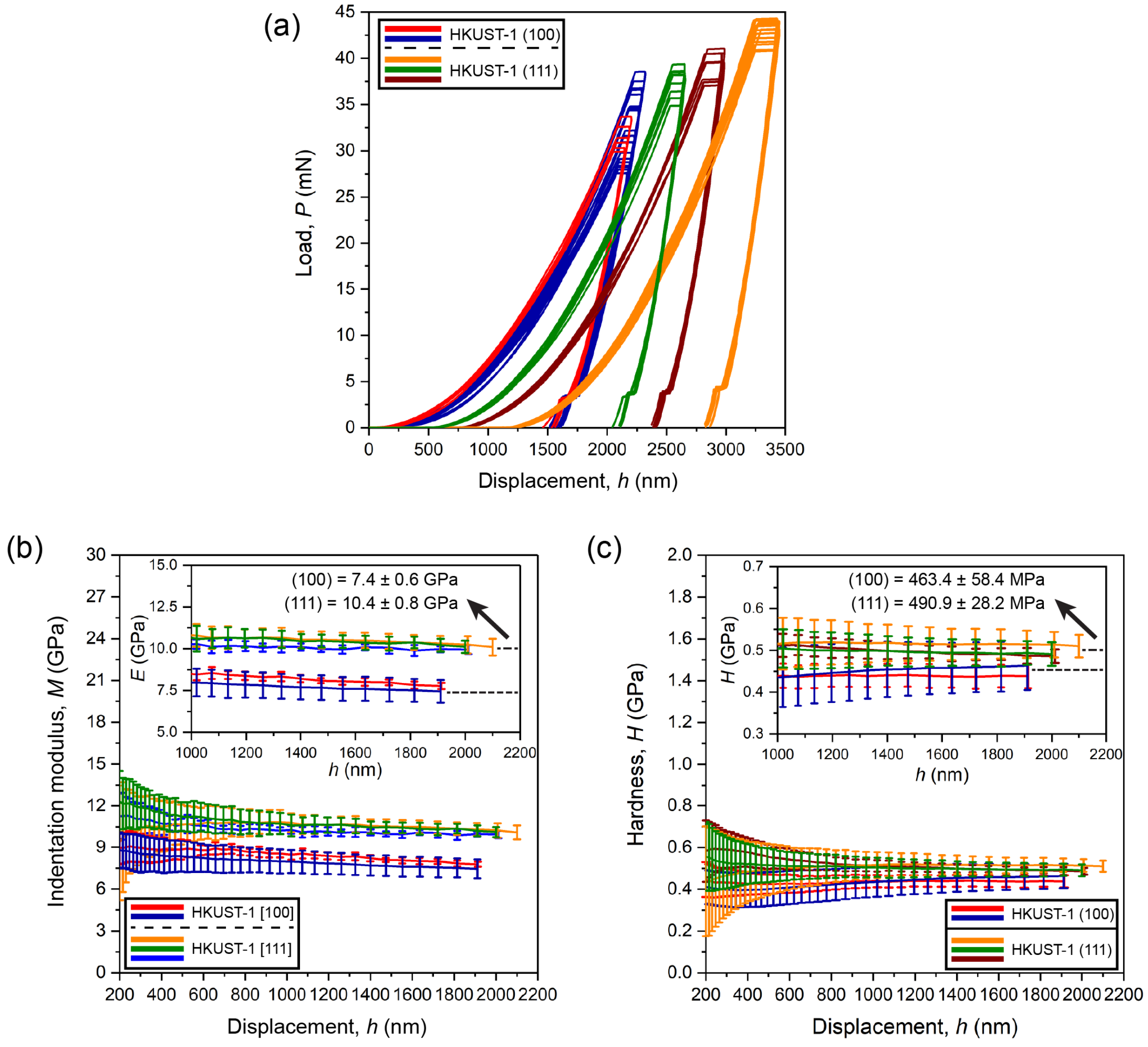

Supplementary Figure 3. Instrumented nanoindentation tests *via* MTS XP using a Berkovich diamond tip. (a) Load vs displacement (*P*-*h*) curves measured from the two HKUST-1 crystal facets. Each test batch represents a set of ~10 measurements. Thermal drift measurements were performed at 90% unload, resulting in some displacements determined at a constant load. (b) Indentation modulus ($M = E/(1 - \nu_s^2)$),[3] and (c) hardness (*H*) data as a function of indentation depth, determined by continuous stiffness measurement (CSM) method. Insets show the average values derived from a surface penetration depth of 1000–2000 nm. The error bars represent the standard deviations.

5. Supplementary Note 2: Yield Stress (*Y*) Determination

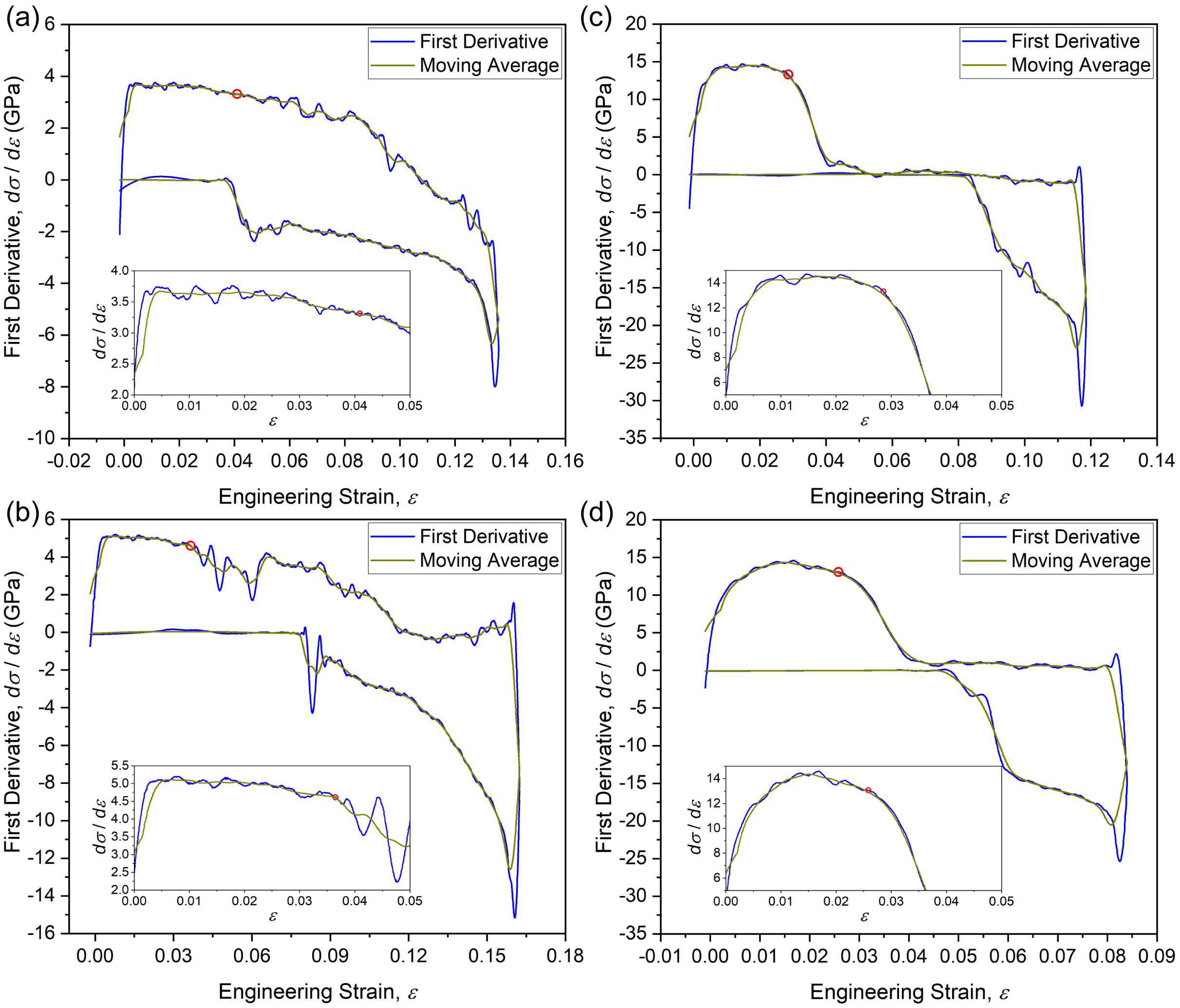

Supplementary Figure 4. First-derivative plots (dσ/dε) of the stress-strain curves, after being smoothed out using the moving average method for the data obtained from the uniaxial compression tests. Results for the (a-b) (100)- and (c-d) (111)-crystal facets. The yield points are highlighted in the red circles on the first-derivative curve, the views of which are enlarged in the corresponding insets.

To determine the yield point in a more rigorous way, we first differentiated the stress-strain ($\sigma-\varepsilon$) curve from the compression test with the Savitzky-Golay filter applied (4th order polynomial order, and points of window ≈ 3-7% of the total number of data points of the loading portion), and then smoothed out the first-derivative $\left(\frac{d\sigma}{d\varepsilon}\right)$ curve using the unweighted

moving average method (the window length was set to be the same after the Savitzky-Golay filter used in the previous step) to highlight the fluctuations and trends. Subsequently, we applied 90% of the largest first-derivative value as the threshold and also indicator to identify the strain at the contact point and the yield point of the original stress-strain curve. The part of the stress-strain curve enclosed by these two points was regarded as the elastic regime, which was then linearly fitted to determine the value of Young's modulus (E). It is worth noting that the elastic limit and the yield point are typically very close, and hence herein we treated them as being coincident.

6. Gamma Coefficient and *H/E* vs. *Y/E Ratios*

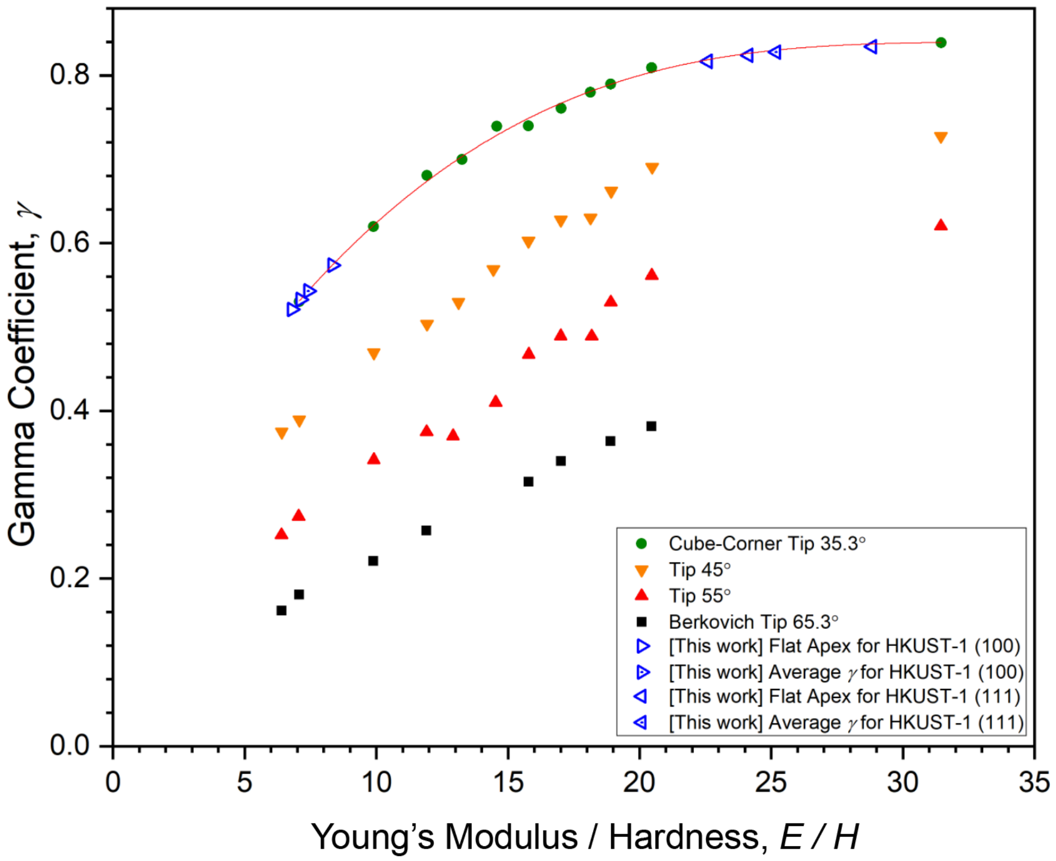

Supplementary Figure 5. Gamma coefficients as a function of the *E*/*H* ratio using the cube-corner indenter to implement the pillar splitting analysis of the two HKUST-1 facets, *viz.* the empty symbols in the figure. Moreover, the gamma coefficients acquired in this work are compared with the values of other materials using indenter tips of different geometries as reported by Ghidelli *et al.*[4] In Ghidelli's work, it should be noted that some of the data points were measured from pillar splitting technique while others were simulated by finite-element modelling (FEM). Adapted from ref. [4] with permission. Copyright (2017) The American Ceramic Society. (b) *H*/*E* vs. *Y*/*E* for the two HKUST-1 facets in comparison with the Tabor and Johnson relations.[5,6]

Supplementary References

1 Wheeler, J. M. & Michler, J. Elevated temperature, nano-mechanical testing in situ in the scanning electron microscope. *Rev. Sci. Instrum.* **84**, 045103 (2013).

2 Howatson, A. M., Lund, P. G. & Todd, J. D., Engineering Tables and Data (HLT). P. D. McFadden & P. J. Probert Smith, Eds., (Department of Engineering Science, University of Oxford, UK, 2009).

3 Tan, J. C., Merrill, C. A., Orton, J. B. & Cheetham, A. K. Anisotropic Mechanical Properties of Polymorphic Hybrid Inorganic-Organic Framework Materials with Different Dimensionalities. *Acta. Mater.* **57**, 3481-3496 (2009).

4 Ghidelli, M., Sebastiani, M., Johanns, K. E. & Pharr, G. M. Effects of indenter angle on micro-scale fracture toughness measurement by pillar splitting. *J. Am. Ceram. Soc.* **100**, 5731-5738 (2017).

5 Tabor, D. The physical meaning of indentation and scratch hardness. *Br. J. Appl. Phys.* **7**, 159-166 (1956).

6 Johnson, K. L. The Correlation of Indentation Experiments. *J. Mech. Phys. Solids* **18**, 115-126 (1970).
