# Supplementary material for: In situ micropillar compression of an anisotropic metal-organic framework single crystal
Source: Commun Chem. 2023 Apr 4;6:63. doi: 10.1038/s42004-023-00858-w (PMC10073295; doi:10.1038/s42004-023-00858-w)
Supplement: Supplementary file 3 — Description of Additional Supplementary Files [file 42004_2023_858_MOESM3_ESM.pdf]

Description of Additional Supplementary Files

File name: Supplementary Movie 1

Description: HKUST-1 (100) P1 micropillar compression

File name: Supplementary Movie 2

Description: HKUST-1 (100) P2 micropillar compression

File name: Supplementary Movie 3

Description: HKUST-1 (100) P3 micropillar compression

File name: Supplementary Movie 4

Description: HKUST-1 (100) P4 micropillar splitting

File name: Supplementary Movie 5

Description: HKUST-1 (100) P5 micropillar splitting

File name: Supplementary Movie 6

Description: HKUST-1 (111) P6 micropillar compression

File name: Supplementary Movie 7

Description: HKUST-1 (111) P7 micropillar compression

File name: Supplementary Movie 8

Description: HKUST-1 (111) P8 micropillar compression

File name: Supplementary Movie 9

Description: HKUST-1 (111) P9 micropillar splitting

File name: Supplementary Movie 10

Description: HKUST-1 (111) P10 micropillar splitting
